# Mapping of *Ppd-B1*, a Major Candidate Gene for Late Heading on Wild Emmer Chromosome Arm 2BS and Assessment of Its Interactions with Early Heading QTLs on 3AL

**DOI:** 10.1371/journal.pone.0147377

**Published:** 2016-02-05

**Authors:** Wei Zhou, Shasha Wu, Mingquan Ding, Jingjuan Li, Zhaobin Shi, Wei Wei, Jialian Guo, Hua Zhang, Yurong Jiang, Junkang Rong

**Affiliations:** The Key Laboratory for Quality Improvement of Agricultural Products of Zhejiang Province, School of Agriculture and Food Science, Zhejiang A&F University, Linan, Hangzhou, Zhejiang 311300, China; Department of Agriculture and Food Western Australia, AUSTRALIA

## Abstract

Wheat heading date is an important agronomic trait determining maturation time and yield. A set of common wheat (*Triticum aestivum* var. Chinese Spring; CS)-wild emmer (*T*. *turgidum* L. subsp. *dicoccoide*s (TDIC)) chromosome arm substitution lines (CASLs) was used to identify and allocate QTLs conferring late or early spike emergence by examining heading date. Genetic loci accelerating heading were found on TDIC chromosome arms 3AL and 7BS, while loci delaying heading were located on 4AL and 2BS. To map QTLs conferring late heading on 2BS, F_2_ populations derived from two cross combinations of CASL2BS × CS and CASL3AL × CASL2BS were developed and each planted at two times, constituting four F_2_ mapping populations. Heading date varied continuously among individuals of these four populations, suggesting quantitative characteristics. A genetic map of 2BS, consisting of 23 SSR and one single-stranded conformation polymorphism (SSCP) marker(s), was constructed using these F_2_ populations. This map spanned a genetic length of 53.2 cM with average marker density of 2.3 cM. The photoperiod-sensitivity gene *Ppd-B1* was mapped to chromosome arm 2BS as a SSCP molecular marker, and was validated as tightly linked to a major QTL governing late heading of CASL2BS in all mapping populations. A significant dominance by additive effect of *Ppd-B1* with the *LUX* gene located on 3AL was also detected. CS had more copies of *Ppd-B1* than CASL2BS, implying that increased copy number could elevate the expression of *Ppd-1* in CS, also increasing expression of *LUX* and *FT* genes and causing CS to have an earlier heading date than CASL2BS in long days.

## Introduction

Heading date is an important agronomic trait that has attracted considerable attention in bread wheat (*Triticum aestivum*, 2n = 6x = 42, AABBDD) breeding programs. It affects wheat maturation time, and further influences yield potential and quality [1]. Wheat flowering time is complex, exhibiting continuous variation among different varieties. Photoperiod (*Ppd*), vernalization (*Vrn*) response, and earliness *per se* (*eps*) genes are three important factors involved in regulating wheat flowering time [2–4].

Vernalization is the acquisition of the ability to accelerate flowering following exposure to cold or a chilling treatment; response to vernalization is dependent on the intensity of low temperature and the duration of exposure [5]. Genetic differences in vernalization requirement are mainly caused by allelic variation at the loci *Vrn*-1, *Vrn*-2, *Vrn*-3, and *Vrn*-4. *Vrn*-1 series genes consist of three separate orthologous counterparts (*Vrn*-A1, *Vrn*-B1, and *Vrn*-D1), located on chromosomes 5A, 5B, and 5D, respectively [6–9]. Among *Vrn*-2 series genes, *Vrn*-A2 and *Vrn*-B2 have been characterized in diploid and tetraploid wheat [2,10]. Distelfeld et al. [2] demonstrated that *Vrn*-B2 is generally functional whereas *Vrn*-A2 is non-functional in tetraploid wheat. The *Vrn*-3 series includes *Vrn*-A3, *Vrn*-B3, and *Vrn*-D3 loci in hexaploid wheat [11], mapped on chromosomes 7A, 7B, and 7D, respectively [10,12]. In most wheat varieties, *Vrn*-1 is up-regulated by either vernalization or by mutations in the *Vrn*-1 gene itself. *Vrn*-1 activates expression of *Vrn*-3 (*FT1*) by binding directly to the promoter of *Vrn*-3 (*FT1*) and precedes long-day induction of *Vrn*-3 [10,13]. As yet, only *Vrn*-D4 in *Vrn*-4 alleles has been described, mapping on chromosome 5D [14].

Photoperiod response is an important genetic system determining flowering time and adaptation of wheat to different agro-climatic conditions. Photoperiod response genes play a key role in accelerating or delaying heading time under field conditions in spring-sown cultivars not sensitive to low temperature [15]. According to their response to day length, wheat varieties are mainly classified into two types: photoperiod insensitive (PI) and photoperiod sensitive (PS). PI varieties have a short delay in time to flowering while PS varieties display a significant delay in flowering under short day (*SD*) conditions (10 h light or less), provided that any vernalization requirements have been met. PI is mainly determined by *Photoperiod-1* (*Ppd-1*) genes located on chromosomes 2A (*Ppd-A1*), 2B (*Ppd-B1*), and 2D (*Ppd-D1*) [4]. *Ppd-1* genes are members of the pseudo-response regulator (*PRR*) family, orthologous to the *Ppd-H1* gene of barley (*Hordeum vulgare*, 2n = 2x = 14, HH). *Ppd-H1* has a CCT domain showing high similarity to *Arabidopsis PRR7* [16,17], a gene with known circadian clock function [18]. When three wheat *Ppd-1* members were individually introduced into the U.K. winter wheat variety ‘Mercia’, lines carrying *Ppd-D1* were the earliest to flower under greenhouse conditions, those carrying *Ppd-B1* the latest, and those with *Ppd-A1* intermediate [15]. Therefore, *Ppd-D1* confers a stronger effect on heading date than the other two *Ppd-1* homologs [7,15]. Studies in hexaploid wheat revealed that a semi-dominant mutation is present in upstream of the coding region of the PS allele *Ppd-D1b*. A 2089 bp deletion mutation in *Ppd-D1b* created the pseudo response regulator (PRR) gene (*Ppd-D1a*); this converts PS cultivars into PI cultivars [17]. Similarly, two *Ppd-A1a* alleles, with 1,027 bp or 1,117 bp deletions at the upstream coding region, are also associated with photoperiod insensitivity [19]. In contrast to *Ppd-A1* and *Ppd-D1*, no sequence insertions and/or deletions are present in *Ppd-B1a* and *Ppd-B1b* [17]. Previous studies also did not reveal any mutation in the *Ppd-B1* gene responsible for flowering in commercial wheat varieties. However, genetic and genomic approaches illustrated that the alleles alter flowering time through increasing gene copy number and further modifying gene expression [20].

When environmental requirements for vernalization and photoperiod have been fulfilled, different wheat varieties still flower at different times even growing in the same environment. Such genetic differences are termed ‘earliness *per se*’ (eps) [21,22]. Photoperiod and vernalization response genes control flowering time of wheat in response to specific day length and temperature, whereas *eps* genes affect flowering time independent of environmental stimuli [1]. Genetic analyses indicate that *eps* genes have relatively smaller effects on flowering time than vernalization or photoperiod response genes, and are generally mapped as quantitative trait loci (QTL) rather than as major genes [23,24]. Since discovery of the first *eps* gene on the long arm of chromosome 2B by Scarth and Law [3], almost all wheat chromosomes have been found to carry *Eps* genes/QTL. They are most frequent on chromosomes 3A [21], 5A [23–26], 2B [3,27–29], 5B, 7B, and 4D [21, 30,31].

Numerous studies have revealed that common wheat, both hexaploid and tetraploid, has relatively narrow genetic variations. This not only increases vulnerability to environmental stresses but seriously limits wheat productivity and end-use quality [32–35]. Wild emmer wheat (*Triticum turgidum* L. subsp. *Dicoccoides* (TDIC), 2n = 4x = 28; genomes BBAA) is the progenitor of durum wheat and the donor of the A and B genomes of hexaploid wheat. It potentially contains novel alleles that would be useful in modern breeding programs, such as grain yield, grain protein, disease resistance and tolerance to stresses [36–40].

Chromosome engineering was used to create a set of chromosome arm substitution lines (CASLs) of wild emmer line TDIC140 in the background of common wheat cultivar ‘Chinese Spring’ (CS) [39–41]. The present study employed a field experiment to examine heading date variation among these CASLs. The heading dates of CASLs 4AL and 2BS were 21.4 and 13.8 days later than CS, respectively, while those of CASLs 3AS, 7BS, and 3AL were 4.6, 6.2, and 8.4 days earlier than CS. Use of F_2_ populations derived from crosses between CASL2BS and CS or CASL3AL revealed that an important heading date related QTL was consistently mapped on chromosome 2BS. Based on locations corresponding with the QTL, *Ppd-B1* was deduced to be the best candidate gene for the late heading of CASL2BS.

## Materials and Methods

### Plant materials and mapping populations

The materials used in this study included 28 CASLs and their parents (TDIC140 and CS, Table 1), which were kindly provided by Dr. Feldman at the Weizmann Institute of Science, Israel. TDIC140 is a wild emmer accession collected in its primary habitat between Rosh Pinna and Zefat, Eastern Galsee, Israel and has high grain protein content and large grains [37, 39]. These CASLs were developed by Feldman et al. [37] through crosses between TDIC and CS DT lines and seven backcrosses of the resultant offspring to corresponding DT lines, coupled with cytogenetic verification of the chromosome constitution in each generation [37, 39]. In each CASL, a pair of targeted TDIC chromosome arms was confirmed by SSR markers as having replaced its homologous CS pair [40]. To determine the QTLs for later heading date of CASL2BS, two cross combinations of CASL2BS × CS and CASL3AL ×CASL 2BS were made to develop F_2_ mapping populations, which were planted at two sowing times (November 10, 2012 and January 26, 2013) forming four F_2_ segregating populations named E1-E4 (Table 2).

**Table 1:** Heading date variation and significance analyses of 28 CASLs and *Triticum aestivum* var. Chinese Spring (CS).

CASLs and CS	Average heading date value (d)	^†^*P* = 0.01 Significance	^‡^ SD (d)
4AL	179.2	A	1.4
2BS	171.6	B	2.3
6BL	159.6	C	0.5
6BS	159.4	CD	0.5
5BL	158.6	CDE	1.7
7BL	158.6	CDE	1.5
5BS	158.4	CDEF	1.1
3BL	158.2	CDEF	1.8
4AS	158.2	CDEF	1.8
6AS	158.0	CDEF	0.8
CS	157.8	CDEF	1.4
2AL	157.8	CDEF	1.0
1AS	157.6	CDEF	0.9
5AL	157.6	CDEF	0.9
1AL	157.4	CDEF	1.9
7AL	157.2	CDEF	0.6
1BS	156.8	CDEFG	1.4
4BS	156.6	CDEFG	2.7
7AS	156.4	CDEFG	1.5
1BL	156.0	CDEFG	1.6
2BL	155.6	CDEFG	1.0
2AS	155.6	CDEFG	0.9
5AS	155.4	DEFG	2.7
6AL	155.0	EFG	1.2
4BL	154.8	EFG	1.8
3BS	154.4	FG	1.1
3AS	153.2	G	1.0
7BS	151.6	G	1.3
3AL	149.4	G	1.4

^**†**^ CASLs sharing the same letter are not significantly different in heading date at the 0.01 probability level, as revealed by Duncan’s multiple comparison.

^‡^ Standard deviation was used to evaluate average heading date.

**Table 2:** General heading date statistics for the four F_2_ populations and their two parents.

Populations	Plant time	Parents (Mean±SD)		F_2_ population		One-sample t-test
		CASL2BS	CS/CASL3AL	Mean±SD	Range	CASL2BS
CASL2BS × CS (E1)	11/10/2012	171.2±1.3	154.3±1.2	160.6±2.1	154–171	12.00**
CASL2BS × CS (E2)	1/26/2013	130.4±1.5	117.6±1.7	120.5±1.8	117–129	23.31**
CASL3AL × CASL 2BS (E3)	11/10/2012	171.4±2.1	150.6±1.1	158.8±2.2	149–173	10.18**
CASL3AL × CASL 2BS (E4)	1/26/2013	130.2±1.8	113.6±1.8	119.2±1.6	113–130	6.59**

** Significant at *P <* 0.001

### Field experiment and statistical analysis

Twenty-eight CASLs and their parents (CS and TDIC140) were planted in our university farm at normal sowing season (November 10th) of 2012 using a randomized block design to evaluate heading date. About 10 seedlings of each genotype were planted at spacing of 15 cm in a row, with rows 40 cm apart and 1.5 m in length. Each genotype was repeated five times. F_2_ plants were also planted at spacing of 15 cm in a row, with rows 20 m long. The heading date of each F_2_ plant was recorded when 50% of the ears emerged from their flag leaves. Duncan’s multiple comparisons were used as a measurement of significance among CASLs and between CASLs and CS (*P* = 0.01).

### DNA extraction and molecular marker analysis

Approximately 0.2 g fresh leaf tissue was collected for DNA extraction by the cetyl trimethyl ammonium bromide method (CTAB) described by Khavkin and Coe [42]. To map the *Ppd-B1* gene, its conserved sequence was used to design primers (forward: 5’-GTTACTATCTCTCATGGTGTAT-3’ and reverse: 5’-AGCAGTTTCCTCGTACAGTTTA-3’) for development of a corresponding SSCP (Single-Strand Conformation Polymorphism) marker. A total of 45 SSR markers were first selected from the published genetic maps of chromosome 2BS to screen for polymorphisms between the two parents (CS and TDIC140) [43–48]. All primer pairs were synthesized by Shanghai Sangon Biotechnology Company Limited. The PCR mixture contained 50 ng DNA, 2.5 mM MgCl_2_, 1.5 mM deoxyribonucleotide triphosphate, 1.5 μL 10x PCR buffer (Sangon, Shanghai, China), 1.5 μM of each primer, and 1 U *Taq* DNA polymerase in a total volume of 15 μL. The PCR conditions varied slightly among different primers according to their annealing temperature, but most use cycling conditions similar to: 3 min at 95°C followed by 30 cycles of 45 s at 94°C, 40 s at the optimized annealing temperature, and 45 s at 72°C. After cycling, the reactions were incubated at 72°C for 7 min. The PCR products were separated with 8% polyacrylamide gels. Gels were electrophoresed at constant voltage (180 V) for 1h and processed with a silver staining method described by Bassam et al. [49]. SSCP analysis of *Ppd-B1* was carried out as reported [50].

### Linkage map construction

Linkage analysis was performed using MapMaker/exp Version 3.0. The Kosambi mapping function [51] with a minimum LOD (Logarithm of the odds) threshold of 3.0 and a linkage threshold significance of *P* = 0.05 was applied to transform recombination frequencies into genetic distances in centiMorgans. Linkage groups were assigned to chromosomes based on the published genetic maps (available at the Grain Genes website at http://wheat.pw.usda.gov).

### QTL analysis on chromosome arm 2BS and estimation of epistatic effects

QTLs were detected with the WinQTLCart2.5 software by composite interval mapping (CIM) [52,53] using the Zmapqtl model 6 with a window size of 10 cM and a 1 cM walk speed. The statistical significance thresholds used to declare the presence of QTLs were determined by 1,000 random permutations with a genome-wide type I error rate of 5% [54]. The 90% likelihood interval of QTL locations was determined by one LOD intervals surrounding the QTL peak [55].

The epistatic and additive effects among QTLs were examined with QTL Network 2.0 [56,57]. The 1D search for the main effect QTL was done with a 10-cM testing window, 1-cM walking speed, and 10-cM filtration window. The total effects of two examined QTLs must have a *P* value less than 1×10^−5^ and interaction effects must have a *P* value less than 0.01 to be deemed significant.

### Copy number analysis of Ppd-B1 by TaqMan^®^ assays

TaqMan assays used 20 μL reactions comprising water (6.4 μL), Passive Reference Dye (50X, 0.4μL) TransStart^®^ Probe qPCR SuperMix (10μL), Probe plus primers (10μM, 0.4μL) and DNA (2μL). Samples were denatured at 95°C for 3 min followed by 40 cycles of [95°C for 15 sec, 57°C for 20 sec, 72°C for 30 sec]. Forward and reverse primers for *Ppd-B1* were gcgtaagttactatctctcatggtgtatc and tttgttttagtacccagtaccataccag (0.2 μM each) and the probe sequence was FAM-ctgctgcttcagttcctagtttcacttgtgtcc-BHQ1(0.2 μM). Forward and reverse primers for the *TaCO2* control were tgctaaccgtgtggcatcac and ggtacatagtgctgctgcatctg (0.2 μM each) and the probe sequence was FAM-catgagcgtgtgcgtgtctgcg-bhq1 (0.2 μM). *Ppd-B1* was analyzed together with the control in multiplexed reactions [20].

### Time-course RT-qPCR

RNA was extracted from seedlings of CS, TDIC140 and CASL2BS, grown in a controlled environment room with short day treatment (15 hrs light at 16°C and 9 hrs dark at 14°C) for 30 days after germination. Leaves were harvested every 3 hrs over a 24 hr period from at least three plants per time point per genotype (biological replicates) and immediately frozen in liquid nitrogen. RNA extraction was performed by using PureLinkRNA mini kits (Invitrogen) in combination with TRIzol reagent (Invitrogen) as described by the manufacturer. DNA was removed by DNaseI digestion. cDNA was synthesized with Superscript II (Invitrogen) using the manufacturer’s protocols with 5μg of total RNA as template and a mixture of Oligo dT(12–18) (250 ng) and random hexamers (150 ng) as primers. Real-time PCR used 1/40 by volume of the final cDNA aliquot as described below. Primer pairs for time course expression of clock genes including *Photoperiod-1* (*Ppd-1*), *FLOWERING LOCUS T* (*FT*), *LUXARRHYTHMO* (*LUX*) were as follows; *Ppd-1* specific primers: agacgattcattccgctcc and agcagcaccatttgacagg. *FT* specific primers: gcaggaggtgatgtgctacgag and aggttgtagagctcggcgaagt. *LUX* specific primers: cggctacggttttgacttcggg and ccctgcatccgcttgacgtagag [58].

## Results

### Variation of heading dates regulated by different TDIC chromosome arms

The 28 CASLs and the recipient parent CS grown in the field showed obvious and statistically significant variations in heading date. CASLs 3AS, 3AL, 4AL, 2BS, and 7BS displayed the most significant differences in heading date compared with CS (Table 1). There was approximately 1 month’s difference in heading date between the earliest (3AL) and latest (4AL). CASLs 4AL and 2BS started ear emergence about 21.4 and 13.8 days later than CS, respectively, while CASLs 3AS, 7BS, and 3AL were 4.6, 6.2, and 8.4 days earlier than CS (Table 1). This suggests that genetic loci accelerating heading of CS were located on chromosomes 3AS, 3AL and 7BS of wild emmer, with loci on chromosome arms 4AL and 2BS delaying heading of CS.

### Segregation analysis of heading date in the F_2_ populations

**Fig 1:**
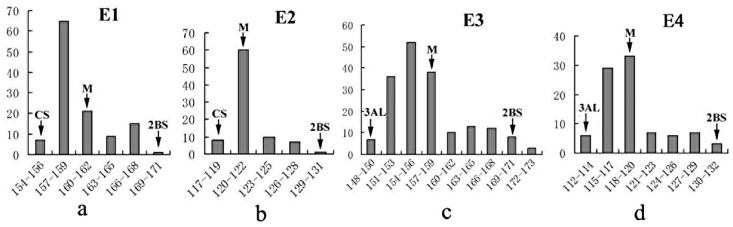
Distribution of heading dates in four wheat F_2_ populations. M: Middle value; CS: Chinese Spring; 2BS: CASL2BS; 3AL: CASL3AL; E1:CASL2BS×CS (Planting time: 11/10/2012); E2: CASL2BS×CS (Plant time:1/26/2013); E3: CASL3AL×CASL 2BS (Planting time: 11/10/2012); E4:CASL3AL×CASL 2BS (Planting time: 1/26/2013).

CASL2BS started ear emergence about 13.8 days later than CS, while CASL3AL was about 8.4 days earlier than CS when planted at the normal sowing time (Nov. 10, 2012) in Zhejiang, China. When sowed in the cold season (January 26, 2013), heading dates of these lines were about 40 days less than when grown at the normal winter sowing time.CS and CASL3AL were crossed with CASL2BS to map QTLs related to later heading on TDIC chromosome arm 2BS. All tested F_2_ mapping populations displayed continuous variation in heading date. No transgressive segregation was found in three of the four populations, with maximum and minimum heading dates not significantly different from those of their corresponding parents. In population E3, CASL2BS was a little earlier than the latest F_2_ plants. F_2_ populations of two cross combinations showed a similar segregation pattern for heading date (Fig 1) when planted at two different times, though the average heading date of the later plantings (E2 and E4) was much earlier than the earlier plantings (E1 and E3), approximately 120 vs. 160 days (Table 2). The heading dates of the four F_2_ populations showed a continuous distribution, but skewed toward the two earlier heading parents, CS and 3AL. A Shapiro-Wilk test confirmed that distributions were all non-normal (P < 0.001), suggesting that alleles controlling heading date located on CS chromosome 2BS might dominate those of TDIC140. Using the middle heading date as the dividing point, most F_2_ plants in all four populations were classified as having earlier heading (Table 3). The ratios of F_2_ plants in ‘earlier’ versus ‘later’ groups did not deviate significantly from 3:1 segregation (E1: χ^2^_0.05, (1)_ = 0.19; E2: χ^2^_0.05, (1)_ = 0.18; E3: χ^2^_0.05, (1)_ = 0.15; E4: χ^2^_0.05, (1)_ = 0.30<χ^2^_0.05, (1)_ = 3.84), indicating that a dominant allele on 2BS might be responsible to early heading.

**Table 3:** Segregation ratio analysis of the four F_2_ populations.

Populations	No. of F_2_ population	Phenotypic segregation ^a^		Expected ratio	χ^2^ b
		Early heading	Late heading		
CASL2BS ×CS (E1)	110	85	25	3:1	0.19
CASL2BS ×CS (E2)	89	69	20	3:1	0.18
CASL3AL×CASL2BS(E3)	179	137	42	3:1	0.15
CASL3AL×CASL2BS(E4)	91	71	20	3:1	0.30

^a^ Earlier than the mean of the earliest and latest heading date was counted as the early heading phenotype; the reverse was counted as the late heading phenotype.

^b^ χ^2^_0.05, (1)_ = 3.84

### Linkage map construction of chromosome arm 2BS

**Fig 2:**
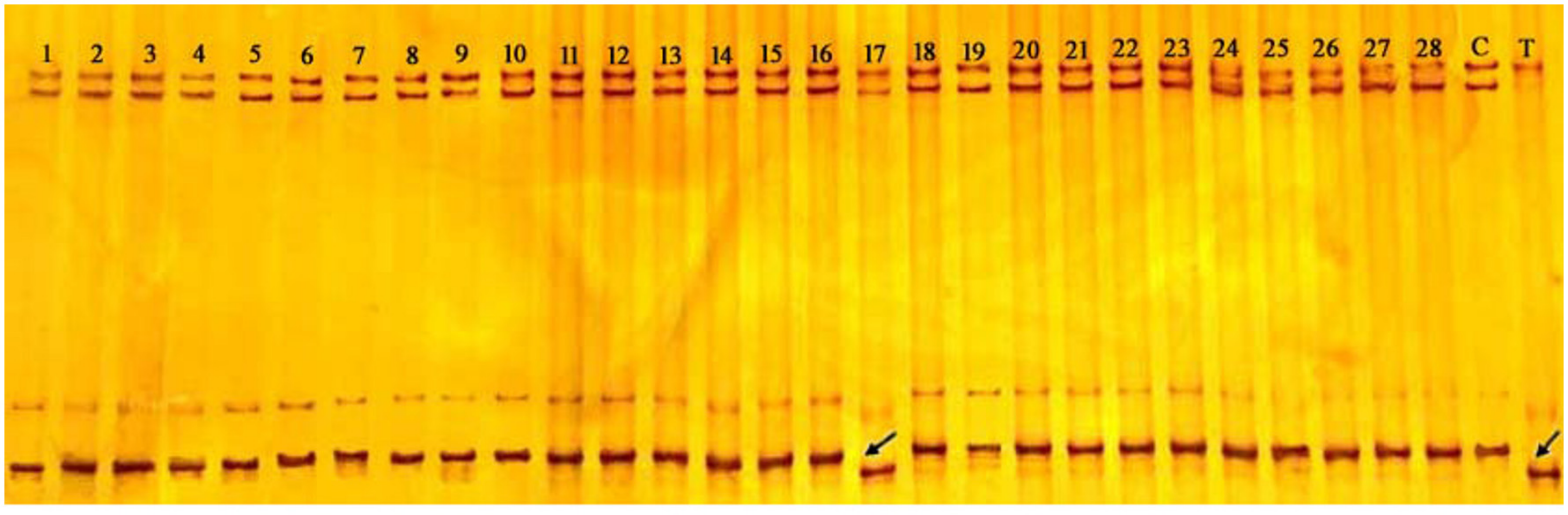
Identification of the *Ppd-B1* gene on chromosome arm 2BS by SSCP analysis of 28 CASLs (#1-#28) and their parents. C: CS; and T: TDIC140. The black arrow indicates the TDIC band in CASL2BS (#17).

**Fig 3:**
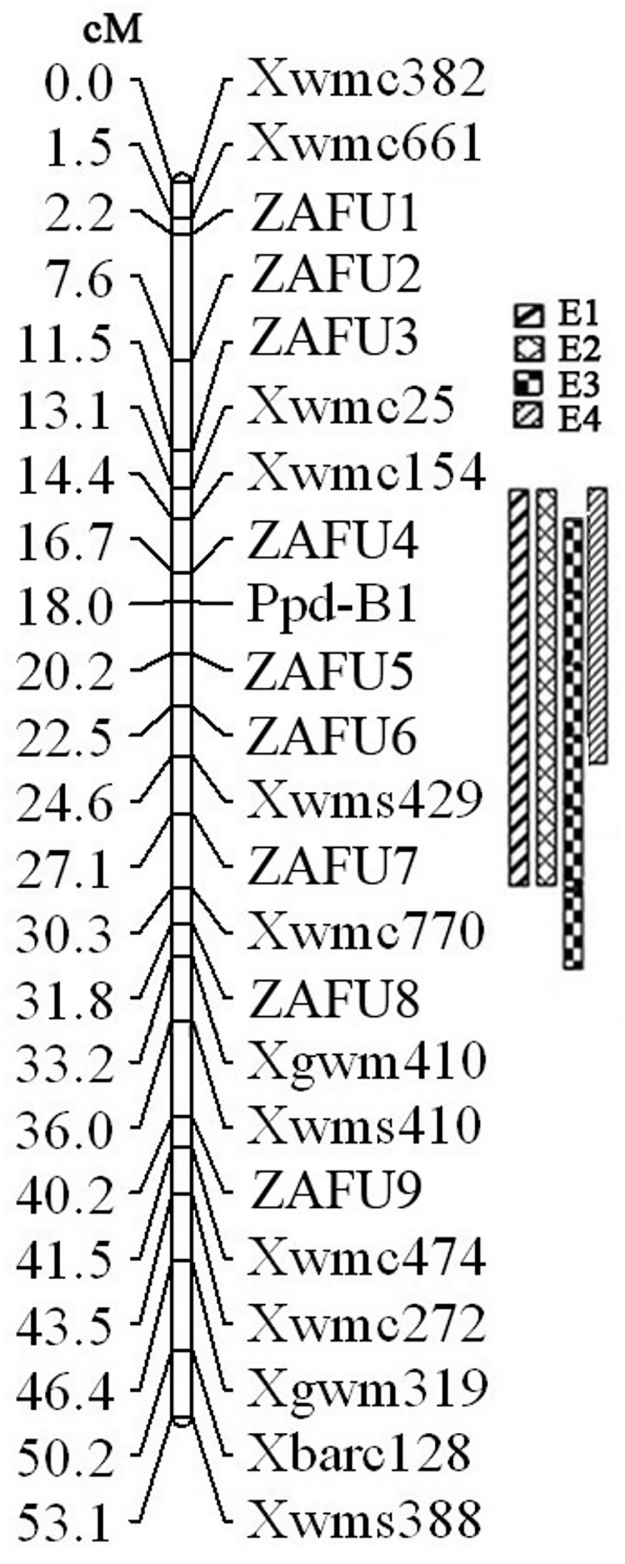
Linkage map of wheat chromosome 2BS constructed using segregation data from four F_2_ populations and locations of QTL related to heading date.

Among forty-five SSR primer pairs previously mapped on chromosome arm 2BS [40,43,46], 13 (28.9%) displayed polymorphism between the two parents (CS and CASL2BS). To add to the density of the genetic map, an additional 120 SSR primer pairs located in the interval between *Xwmc661* and *Xbarc128* according to the published whole genome sequences [40,43,46] were developed and named with our university abbreviation (ZAFU) followed by numbers for different primers. Among these 120 SSRs, only 9 (7.5%) showed polymorphism between the two parents (S1 Table). A *Ppd-B1* primer pair, designed based on conserved regions of the *Ppd-B1* gene, also detected polymorphism between CS and TDIC by the SSCP method [50]. The whole set of CASLs was screened using this marker, with only CASL2BS showing the same amplification band as TDIC140 (Fig 2), confirming that the *Ppd-B1* gene is located on chromosome arm 2BS as reported by Laurie [4]. All F_2_ plants from these four populations were combined to construct a linkage map of chromosome 2BS, including 23 DNA markers spanning 53.1 cM with an average distance between markers of 2.3 cM (Fig 3). *Ppd-B1* was mapped between *ZAFU4* and *ZAFU5* at distances of 1.3 and 2.2 cM, respectively.

### QTL analysis on chromosome arm 2BS and estimation of interactions with QTL on 3AL

An important QTL related to ear emergence was consistently detected in four mapping populations with large LOD scores at an interval of 3.5 cM between *ZAFU4* and *ZAFU5* (Table 4, Fig 3). This indicated a key gene exists on 2BS involved in regulating the ear emergence of CASL2BS, and its function is not significantly affected by the range of environmental conditions in this study. In this QTL region, the *Ppd-B1* gene locus explained the largest portions of phenotypic variation, of 57.24% and 76.72% in E1 and E2 F_2_ populations, with LOD scores of 15.63 and 19.09 respectively. *Ppd-B1* explained lower portions of phenotypic variation in E3 and E4 populations from CASL3AL × CASL2BS, at 23.64% and 29.31% with similarly large LOD scores of 9.96 and 15.77 respectively (Table 4). Therefore, *Ppd-B1b* of TDIC140 is a critical allele for the later heading of CASL2BS.

**Table 4:** QTL locations, related maximum LOD values and their explanation of phenotypic variation for heading date detected in the four mapping populations.

Populations	Marker interval (cM, LOD > 3)	Closest marker to QTL	LOD value	Phenotypic variation (%)	Additive effect	Positive parent
CASL2BS×CS (E1)	*Xwmc25-Xwmc770* (13.1–30.3)	*Ppd-B1*	15.63	57.24	3.56	CS
CASL2BS×CS (E2)	*Xwmc25-Xwmc770* (13.1–30.3)	*Ppd-B1*	19.09	76.72	3.06	CS
CASL3AL×CASL2BS (E3)	*Xwmc154- Xgwm410* (14.4–33.2)	*Ppd-B1*	9.96	23.64	3.98	3AL
CASL3AL × CASL2BS (E4)	*Xwmc25-Xwms429* (13.1–24.6)	*Ppd-B1*	15.77	29.31	5.82	3AL

The QTL Network 2.0 program was used to detect interactions among QTLs conferring later and earlier heading in the CASL3AL × CASL2BS F_2_ population. A significant (*P*<0.05) dominance by additive effect was observed between the *Ppd-B1a* locus on chromosome arm 2BS and *LUX* locus on 3AL(S1 Fig), with an estimated dominance by additive value of 1.92[58].

### Copy number variation (CNV) of *Ppd-B1* and expression analysis of flowering genes

**Fig 4:**
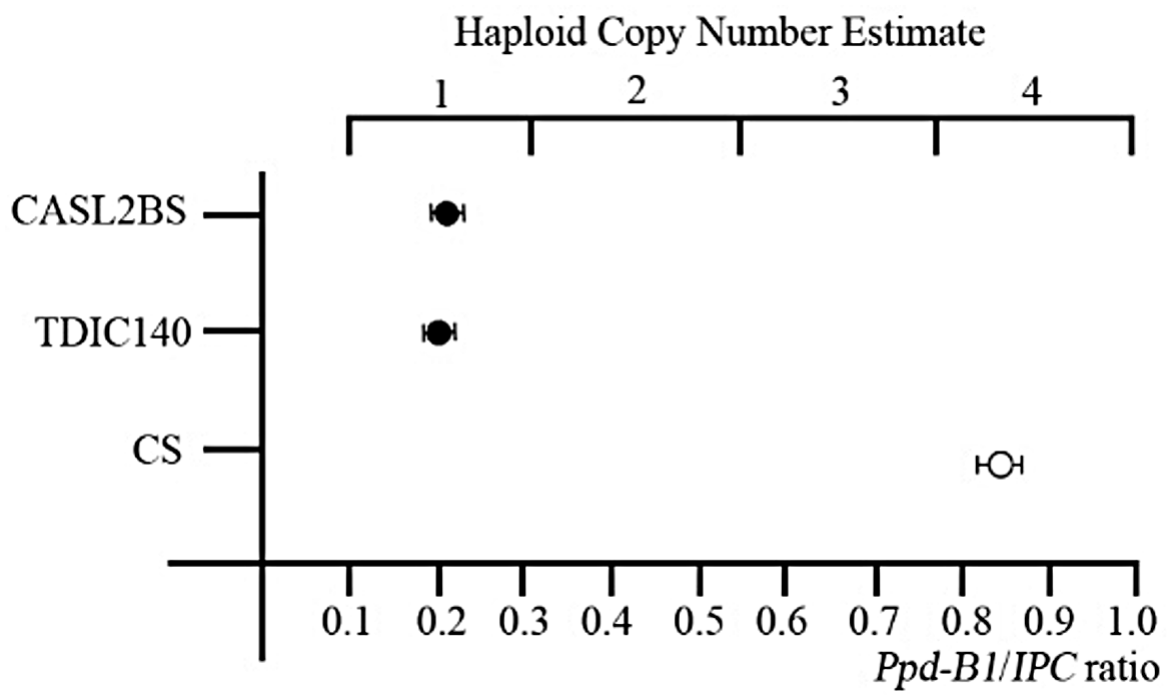
TaqMan^®^ estimation of *Ppd-B1* copy number in Chinese Spring (CS), TDIC140 and CASL2BS. Solid circles are genotypes known to have a photoperiod sensitive (*Ppd-B1b*) allele and open circles are genotypes known to have a day neutral (*Ppd-B1a*) allele. Copy number was estimated based on the *Ppd-B1*/Internal Positive Control (IPC) signal ratio. Means and standard deviations of three measurements are shown. T-tests show the classes that differed significantly from one another (*p<0.001).

To determine copy number of *Ppd-B1* in CS, TDIC140 and CASL2BS, a specific quantitative TaqMan^®^ assay was developed. Part of the wheat CONSTANS2 gene (*TaCO2*, also called *TaHd1* [20]) was used as an internal control. By this assay, TDIC140 and CASL2BS were confirmed to carry one haploid copy (i.e. one copy per 2B chromosome), while CS had four copies (Fig 4).

**Fig 5:**
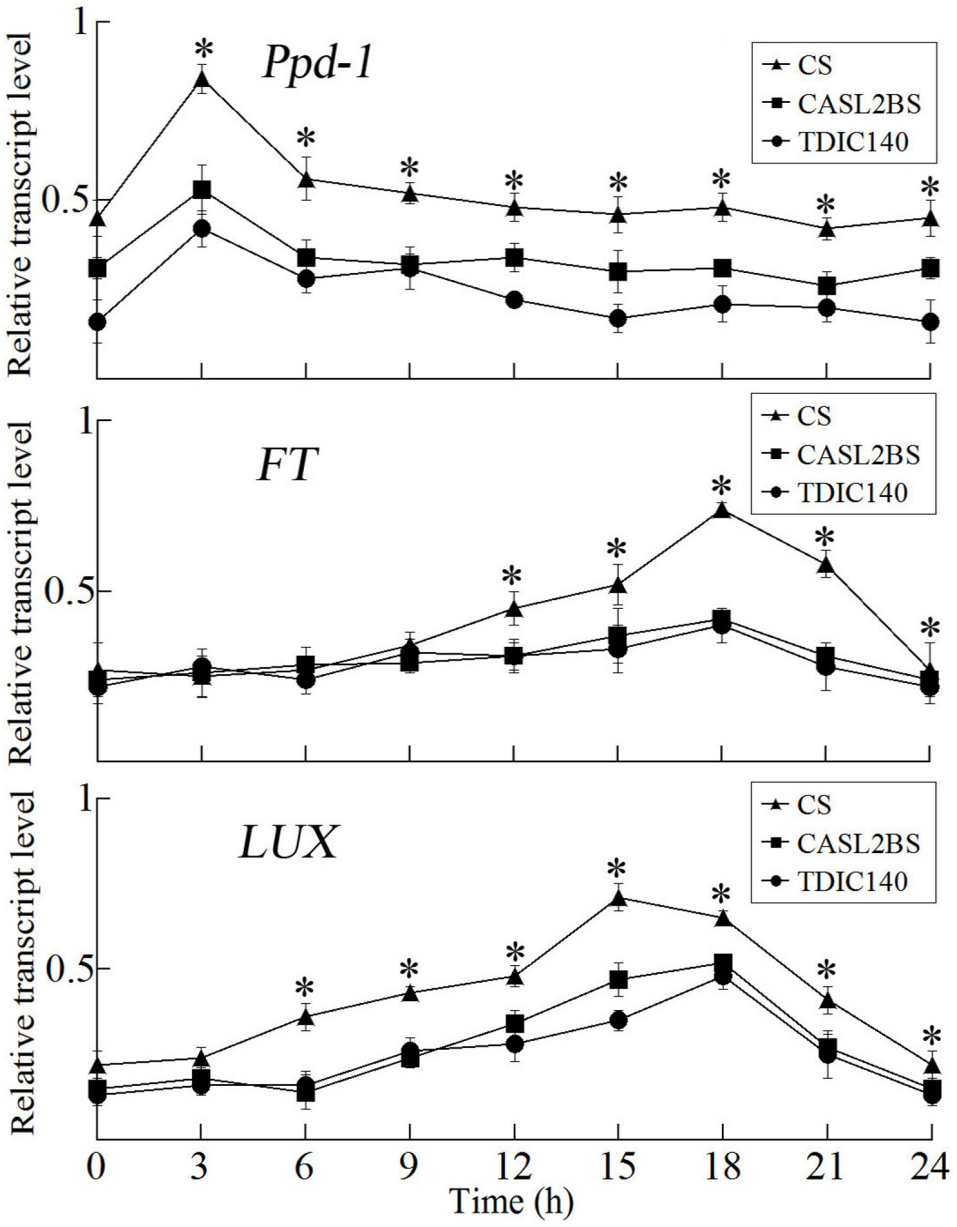
Relative transcript levels of three flowering genes in CS (black triangles), TDIC140 (black diamonds) and CASL 2BS (black squares) using time-course RT-qPCR. Sampling was performed on 30-day-old seedlings starting at 6:00 AM (Time = 0) of the second day under long days (15 h light / 9 h dark). Leaves were harvested every 3 hrs from at least three plants per time point per genotype (biological replicates). Error bars indicate standard error of the mean. A sterisks show probabilities that the respective alleles in CS, CASL2BS and TDIC140 have expression levels that are not significantly different, based upon T tests (* *p*<0.001).

To reveal if increased copy number changed the expression of key flowering genes and further affected the heading time of CS and CASL 2BS, RT-qPCR was applied to analyze their expression per time point per genotype over 24 hrs. The expression of *Ppd-1*, *LUX* and *FT* genes in CS were generally higher than CASL2BS and TDIC140 from dawn (6:00 AM) to night (21:00 PM). In constant sunlight, the expression of *LUX* and *FT* genes gradually increased from dawn (6:00 AM) to night (21:00 PM) in CS, while the *LUX* and *FT* genes in CASL2BS did not change noticeably in expression over the whole time period (Fig 5), suggesting that increased copy number of *Ppd-B1* could enhance the expression of *Ppd-1*. As a result, *LUX* and *FT* genes may be expressed at higher levels in CS than CASL2BS, conferring earlier heading in long days.

## Discussion

### CASLs of TDIC140 in the CS background are powerful genetic tools for mapping qualitative and quantitative traits

Due to its importance in wheat breeding, extensive research about heading date has been carried out, and numerous related QTLs have been mapped on nearly all chromosomes of the three homoeologous groups (A, B, and D) of hexaploid wheat. The main reasons for this are the small effects of each QTL and the effects of environmental factors on heading date. Another important explanation for these phenomena is non-linear interactions among different genes and genetic systems [10,11,59].

Many kinds of mapping populations have been explored in heading date QTL studies, including doubled haploid (DH) lines and single seed descent recombinant inbred lines (RILs) [25–28, 30, 60, 61]. Using RILs, Maccaferri et al. [29] mapped QTLs related to heading date on chromosome arms 2A, 2B, and 7B in tetraploid wheat. As CASLs used in this study each carry only one TDIC140 arm, they can reduce interference from genes on other chromosomes; making them very powerful for locating qualitative genes. By using parallel CASLs with the same TDIC chromosome arm in the common wheat background (Bethlehem), genes of red coleoptile, waxless plant, brittle rachis, and resistance to several powdery mildew races were allocated to chromosomes 7A, 2BS, 3AS and 3BS, and 2BS, respectively [39,41]. In this study, CASLs 4AL, 2BS, 3A, and 7BS were found to have significantly different heading dates from the recipient parent (CS) following examination over 2 years. This suggests that CASLs 4AL, 2BS, 3A, and 7BS carry important genes related to heading date, and will be of great use in mapping QTLs conferring this complicated trait. An important heading date QTL was consistently mapped in the same region in two mapping populations in both years, and provides a good example of the power of CASLs for mapping complex traits. Presently, this set of CASLs is being used to map genes related to heading date on chromosomes 3AL, 4AL, and 7BS.

QTLs are linked to or contain genes associated with small effects on one specific phenotype; these are difficult to map and are further cloned using temporary mapping populations. Inbred lines are stable and powerful for mapping QTLs. For example, use of sub-introgression lines (ILs) allowed the mapping of QTLs governing leaf morphology and yield related traits in tomato [62–64]. The CASLs used in the present study were confirmed as mainly carrying specific chromosomal arms [40], so resemble an IL. A whole chromosome arm is still large for mapping as it likely carries many genes in addition to the one(s) of interest; these may mask or disturb the function of the targeted genes. ILs with smaller TDIC segments may be created by crossing CASLs with their common wheat parent, and then backcrossing the resulting F_1_ population a few times, using common wheat as the recurrent parent. The resulting ILs will prove powerful for gene identification and cloning, and are presently being constructed in our laboratory.

### Probable mechanism of the *Ppd-B1* gene in regulating flowering time

Different wheat germplasm has varied responses to day length, some being very sensitive while others are insensitive. These differences in response are mainly determined by genes located on homoeologous group 2 chromosomes [4]. Most wild wheat races are sensitive to day length. TDIC140 is a landrace native to Israel, and shows a sensitive response to day length [65,66]. Compared with CS, genes on TDIC140 chromosome 2BS are more sensitive to day length, leading to the heading date of CASL2BS being later than that of CS.

An important QTL identified in the four F_2_ segregating populations involving CASL2BS could explain much of the heading date variation between CASL2BS and CS, suggesting that a key gene determining heading date is located on chromosome arm 2BS. Consistent with findings reported by Mohler et al.[67], the QTL mapped in this study corresponded to *Ppd-B1* on the genetic map, implying that *Ppd-B1* is the most likely candidate gene for the QTL. There are many explanations for how photoperiod sensitive alleles of *Ppd-1* affect variation of heading time among different wheat varieties. For instance, insertions, deletions, or point mutations in these alleles can all be underlying reasons causing flowering time variation in different wheat varieties [17,19,68,69]. Díaz et al. [20] reported that alleles with increased *Ppd-B1* copy number confer an early flowering day neutral phenotype, indicating that copy number variation plays a critical role in wheat adaptation. We found that CASL2BS had one haploid copy of *Ppd-B1*, while ‘Chinese Spring (CS)’ had four copies. We infer that the later heading of CASL2BS than CS was caused by the *Ppd-B1* copy number difference between them. This study also revealed that increased copy number of *Ppd-B1* has a close relationship with higher expression of flowering gene *Ppd1* from dawn (6:00 AM) to night (21:00 PM) in CS than CASL2BS, which probably cause *FT* and *LUX* (a candidate gene in CS responsible for the earliness *per se* 3 (*Eps-3A*^*m*^) locus on chromosome 3AL [58]) to have higher expression in CS than in CASL2BS, and finally lead CS to have a day neutral and earlier flowering phenotype than CASL2BS. Analysis of Ppd-B1 protein sequences between CS and TDIC140 revealed one AA mutant and one AA deletion locus (S2 Fig). The AA deletion locus was speculated to be a key motif within a phosphoacceptor site phosphorylated by histidine kinase homologues. Whether the AA deletion can affect *Ppd-B1* protein activity and alter flowering time in CASL2BS needs further investigation.

### Complexity of the genetic system regulating flowering time

Some QTLs related to flowering time cannot easily be detected, even when two parents with large differences in flowering time are used to develop the mapping population. For example, differences in flowering time between genotypes that are a consequence of interactions among different genes and genetic systems are difficult to detect as QTLs in some populations. QTLs governed by *Ppd-B1* could consistently be detected in two environments (spring sowing and winter sowing), indicating that *Ppd-B1* was stably expressed.

When the software QTL Network 2.0 was used to investigate interactions among QTLs conferring later and earlier heading, a significant (*P* < 0.05) dominance by additive interaction was observed between a locus on 3AL (probably *LUX*) and the *Ppd-B1* locus on 2BS. The *Ppd-1b* allele influences flowering time by down-regulating the vernalization genes *VRN3/FT* and *TaHd1* under short day conditions [59]. Furthermore, the vernalization gene *Vrn*-3 promotes the transcription of *Vrn*-1 alleles located on different chromosomes and thereby accelerates flowering [10,11]. Genetic interactions affecting flowering time are common in wheat. The strong interaction between genes on chromosome arms 2BS and 3AL reported in this study provides an important addition to the model explaining the complex mechanism of wheat flowering time. Whether other interactions exist between QTLs conferring later heading (4AL) and earlier heading (3AS, 3AL, and 7BS) in wheat requires further study, which is ongoing in our laboratory.

## Supporting Information

S1 Fig Identification of the *LUX-A* gene on chromosome arm 3AL by SSCP analysis of 28 CASLs (#1-#28) and their parents. To map the *LUX-A* gene, its special region between A and B genome was used to design primers (forward: 5’-CGGCGGCTATGGAGGGTACGA-3’ and reverse: 5’-GAGGTGGCTGGCGACGTTCT-3’) for development of a corresponding SSCP marker. C: CS; and T: TDIC140. The black arrow indicates the TDIC band in CASL3AL (#6).
(TIF)

S2 Fig Sequence comparison of *Ppd-B1* proteins between CS, TDIC140 and Ppd-B1a (the day neutral allele, the NCBI accession number is AB646974). Black boxes indicate the sites of variation: one A.A deletion, and one A.A mutation.
(TIF)

S1 Table List of SSR primer pairs. (DOC)
