# Supplementary material for: Mapping of Ppd-B1, a Major Candidate Gene for Late Heading on Wild Emmer Chromosome Arm 2BS and Assessment of Its Interactions with Early Heading QTLs on 3AL
Source: PLoS One. 2016 Feb 5;11(2):e0147377. doi: 10.1371/journal.pone.0147377 (PMC4743932; doi:10.1371/journal.pone.0147377)
Supplement: S1 Table — (DOC) [file pone.0147377.s003.doc]

**S1 Table List of SSR primer pairs**

| Markers | Physical location ( bp ) ^a^ | Forward primer  (5’-3’) | Reverse primer  (5’-3’) |
| --- | --- | --- | --- |
| ZAFU1 | 351477 | GCGTTTCATCTCTTTGTCAT | AGGTTTGTGATTGCTGTTTT |
| ZAFU 2 | 683408 | TGTAAAGCACCATTCACTTG | CACGGACAACATCCTAAAAG |
| ZAFU 3 | 906163 | CAAACTTCTGAGCTTCGACT | AGATCAGAGGAAAGTCATCG |
| ZAFU4 | 1064665 | TTCCCTAAAAAGACATTGGA | CCATTCTCTCTTTCTTGCAT |
| ZAFU 5 | 3841842 | ACAAGGTCCTGTTGTGTTCT | CAGGTCCGTTTAAGTGTCAT |
| ZAFU 6 | 5583928 | GGAGAGGCGATTAATTAGGA | TATACGACTACTGCGCTGAT |
| ZAFU 7 | 6440073 | AGTGGTGTGTCCATCTGTCT | AAATTCGCTTTTGGAACCTA |
| ZAFU 8 | 13890459 | ATTCATGGTGGTACATCTCG | GGAGATTGGAATAACAAGCA |
| ZAFU 9 | 33191861 | AAGAACATCGTACTCGGAGA | TTCATCCTCATCTTCTCGAC |

^a^ The physical distance to marker *Xwmc661* based on the referenced A genome of wheat available at IWGSC (http://www.wheatgenome.org)
